# SICD: Novel Single-Access-Point Indoor Localization Based on CSI-MIMO with Dimensionality Reduction

**DOI:** 10.3390/s21041325

**Published:** 2021-02-13

**Authors:** Yunwei Zhang, Weigang Wang, Chendong Xu, Jie Qin, Shujuan Yu, Yun Zhang

**Affiliations:** 1 College of Electronic and Optical Engineering, Nanjing University of Posts and Telecommunications, Nanjing 210023, China; 1218022904@njupt.edu.cn (Y.Z.); 1219023433@njupt.edu.cn (C.X.); 1218022905@njupt.edu.cn (J.Q.); yusj@njupt.edu.cn (S.Y.); y021001@njupt.edu.cn (Y.Z.); 2 National and Local Joint Engineering Laboratory of RF Integration and Micro-Assembly Technology, Nanjing 210023, China

**Keywords:** channel state information, indoor localization, dimensionality reduction, MIMO, naive Bayes

## Abstract

With the rise of location-based services and the rapidly growing requirements related to their applications, indoor localization based on channel state information–multiple-input multiple-output (CSI-MIMO) has become an important research topic. However, indoor localization based on CSI-MIMO has some disadvantages, including noise and high data dimensions. To overcome the above drawbacks, we proposed a novel method of indoor localization based on CSI-MIMO, named SICD. For SICD, a novel localization fingerprint was first designed which can reflect the time–frequency and space–frequency characteristics of CSI-MIMO under a single access point (AP). To reduce the redundancy in the data of CSI-MIMO amplitude, we developed a data dimensionality reduction algorithm. Moreover, by leveraging a log-normal distribution, we calculated the conditional probability of the naive Bayes classifier, which was used to predict the moving object’s location. Compared with other state-of-the-art methods, the results of the experiment confirm that the SICD effectively improves localization accuracy.

## 1. Introduction

The rapid development of location-based services and the rapidly growing requirements with regard to their applications have greatly facilitated the development of indoor localization. This has brought great changes to our lives. The WIFI fingerprint-based indoor localization method [1,2,3,4] has become an important research topic due to its convenience and low cost. To accurately obtain the indoor location information of a moving object, the WIFI fingerprint-based indoor localization method uses a large number of WIFI signal measurements to establish a fingerprint database in the training stage and obtains the location of the moving object by matching fingerprints in the test stage.

The key to indoor localization technology is obtaining accurate and reliable indoor locations. Channel state information (CSI) is sub-carrier level channel information. It can better describe the influence of the moving object on surrounding signals. Therefore, CSI has been proposed by researchers as a fingerprint for WIFI-based indoor localization in recent years. For example, Wang et al. [5] proposed CSI fingerprint-based indoor localization, which uses a deep learning network to achieve localization. Gao et al. [6] performed device-free wireless indoor localization based on the change of the surrounding CSI signals caused by human behavior. In [7], Yu et al. proposed a stable CSI fingerprint extraction method to achieve indoor localization. However, these methods only use the time–frequency characteristics of CSI to construct fingerprints and ignore the impact of space–frequency characteristics of CSI on localization accuracy.

Fortunately, the indoor localization method based on CSI–multiple-input multiple-output (CSI-MIMO) can overcome the above shortcomings. In order to obtain more reliable and valuable CSI information for high-precision indoor localization, researchers have proposed a variety of localization methods based on CSI-MIMO. Chapre et al. [8] proposed a CSI-MIMO fingerprint localization system with multiple access points, which effectively reduced the mean distance error by utilizing the frequency and space diversity of CSI. In [9], Song et al. proposed an algorithm of CSI-based indoor localization for narrowband IoT (Internet of Things), which uses the CSI of multiple narrowband signal transmitters as fingerprints to achieve indoor localization. In [10], Tian et al. proposed an indoor localization method based on the angle of arrival (AoA) of CSI-MIMO, which has better performance than SpotFi [11]. Although the above-mentioned indoor localization methods based on CSI-MIMO can obtain rich and reliable CSI measurements, all of these methods face some challenges, such as noise, high data dimensions, and complex hardware deployment.

To overcome the above-mentioned shortcomings, we designed the SICD, a novel indoor localization scheme based on CSI-MIMO. In SICD, the localization fingerprint reflecting the time–frequency and space–frequency characteristics of CSI-MIMO under a single access point (AP) was first designed. Then, we developed a dimensionality reduction algorithm to map the high-dimensional CSI-MIMO amplitude data to a low-dimensional space. Finally, we leveraged the log-normal distribution to calculate the conditional probability of the naive Bayes classifier, which was used to predict the moving object’s location. Moreover, in order to verify the performance of SICD we conducted extensive experiments in a real indoor environment.

The SICD scheme makes the following key contributions.

(1) We proposed a method to construct localization fingerprints. The method collects MIMO-based CSI measurements under a single access point (AP), and extracts the amplitude information of the CSI-MIMO to construct a localization fingerprint.
(2) We developed a dimensionality reduction algorithm based on locally linear embedding and low rank. The algorithm can map CSI-MIMO amplitude data from high-dimensional space to low-dimensional space and reduce the data redundancy.
(3) We leveraged the log-normal distribution to calculate the conditional probability of the naive Bayes classifier, which can improve the classification performance of the classifier.
(4) Single AP indoor localization based on CSI-MIMO with a dimensionality reduction method was proposed. The experiments were conducted in the laboratory, and the results show that the proposed method is superior to the state-of-the-art method.

The remaining structure of the paper is demonstrated as follows. The related work of CSI and the truncated nuclear norm is briefly reviewed in the next section. In Section 3, we describe the structure and algorithm of SICD in detail. The simulation settings and experimental results are discussed in Section 4. We summarize the paper and look forward to future work in Section 5.

## 2. Related Work

An indoor localization method based on WIFI signals can be divided into received signal strength (RSS)-based and CSI-based methods, which are briefly reviewed in this section.

### 2.1. RSS-Based Indoor Localization

RSS is MAC (Media Access Control) layer information, which describes the attenuation of wireless signals during the propagation. It has been widely used in indoor localization. RSS-based localization methods mainly include geometric localization and fingerprint localization.

For geometric localization methods, localization is performed by converting RSS measurements into the distance. In [12], Mazuelas et al. proposed a dynamic estimation propagation model which uses trilateration to calculate the location of mobile station. In order to ensure the strong correlation between the actual distance and the RSS measurement distance, Wang et al. proposed a measurement distance calculation method based on tree-ring distance [13]. Wang et al. proposed an algorithm based on filtering technology and an optimization method to reduce measurement error [2]. Furthermore, Carlino et al. proposed an RSS-based distributed cooperative localization method with good robustness in a mixed line-of-sight and non-line-of-sight environment [14].

There are two stages for the localization methods based on fingerprints: the training stage and the test stage. RSS measurements are collected at each location and converted into fingerprints during the training stage. The location is estimated by matching the target fingerprint with the offline fingerprint database during the test stage. In [15], Fang et al. designed a time-insensitive localization system based on RSS, which can reduce the localization error caused by RSS fluctuation. The distance measurement of fingerprint matching is the key factor of fingerprint-based localization. The relationship between location distance and RSS similarity is considered [16]. In order to reduce the impact of environmental changes on localization accuracy, Wang et al. proposed a multi-fingerprint localization method based on subspace and RSS [17].

However, there are some disadvantages of RSS-based localization methods. Firstly, RSS is sensitive to time-varied multipath fading, so it is easily affected by multipath and shadow fading. Secondly, RSS is a kind of coarse-grained channel information that averages the received signal amplitudes, making it difficult to obtain the accurate value.

### 2.2. CSI-Based Indoor Localization

The disadvantages of RSS restrict its applications in the field of indoor localization. CSI is subcarrier-level channel information. It can better describe the influence of the moving object on surrounding signals. Therefore, CSI-based indoor localization has attracted more and more attention. Indoor localization based on CSI was proposed in [18,19]. In [20], Yang et al. compared the difference between CSI and RSSI in indoor localization. In [21], Li et al. proposed a technique that uses CSI to reduce the impact of multipath. Moreover, the software development for extracting CSI has also promoted the research of indoor localization based on CSI [22].

In this paper, we used the space and frequency diversity of a single AP to collect CSI. In order to overcome the high-dimensionality problem of CSI-MIMO data, we proposed a data dimensionality reduction algorithm. We used a Bayesian classifier based on the lognormal distribution for localization.

## 3. Preliminaries

### 3.1. Channel State Information

CSI is fine-grained information from the physical layer, which can reflect the channel characteristics of the wireless communication link. It describes the channel quality between devices of the transmitted and received. When the CSI-MIMO system works on a flat fading channel, the signal collected by the received device is defined as

(1) R=HS+N,

where

(2) $$H={\left[H_{1},\ldots ,H_{i},\ldots ,H_{n}\right]}^{T},$$

***R*** and ***S*** represent the signal vectors of the received and transmitted, respectively. ***H*** and ***N*** represent the matrix of CSI and the vector of Gaussian white noise, respectively. *n* is the index of sub-carriers. T represents transposition of a matrix. The CSI value of the *i*-th sub-carrier is expressed as

(3) $$H_{i}=\left|H_{i}\right|\exp\left\{j∠H_{i}\right\},$$

where $\left|H_{i}\right|$ and $∠H_{i}$ represent the amplitude and phase of CSI of *i*-th sub-carrier, respectively.

### 3.2. Truncated Nuclear Norm

In [23], Hu et al. proposed the idea of truncated nuclear norm (TNN), which holds that the TNN of matrix $Z\in R^{a\times b}$ is equal to the sum of min(a,b)−r minimum singular values. The TNN can be formulated as

(4) $${\left\|Z\right\|}_{r}=\sum_{j=r+1}^{\min\left(a,b\right)}\sigma_{j}\left(Z\right),$$

where ${\left\|Z\right\|}_{r}$ is the truncated nuclear norm and $\sigma_{j}\left(Z\right)$ is the *j*-th minimum singular value of ***Z***.

TNN is non-convex and is very difficult to directly solve (4). Therefore, singular value decomposition (SVD) of ***Z*** is used to approximate the solution. Equation (4) can be rewritten as

(5) $${\left\|Z\right\|}_{r}=\sum_{j=1}^{\min\left(a,b\right)}\sigma_{j}\left(Z\right)-\sum_{j=1}^{r}\sigma_{j}\left(Z\right)=\underset{Z}{\min}{\left\|Z\right\|}_{*}-\underset{CC^{T}=I,DD^{T}=I}{\max}\mathrm{tr}\left(CZD^{T}\right),$$

where

(6) $$C={\left(u_{1},\ldots ,u_{r}\right)}^{T},$$

(7) $$D={\left(v_{1},\ldots ,v_{r}\right)}^{T},$$

(8) $$\begin{array}{cc} \mathrm{tr}\left(CZD^{T}\right) & =\;\mathrm{tr}\left({\left(u_{1},\ldots ,u_{r}\right)}^{T}U\Sigma V^{T}\left(v_{1},\ldots ,v_{r}\right)\right)\\ & =\;\mathrm{tr}\left(\left({\left(u_{1},\ldots ,u_{r}\right)}^{T}U\right)\Sigma \left(V^{T}\left(v_{1},\ldots ,v_{r}\right)\right)\right)\\ & =\;\mathrm{tr}\left(\left(\begin{array}{cc} I_{r} & 0\\ 0 & 0 \end{array}\right)\Sigma \left(\begin{array}{cc} I_{r} & 0\\ 0 & 0 \end{array}\right)\right)\\ & =\;\sum_{j=1}^{r}\sigma_{j}\left(Z\right), \end{array}$$

${\left\|Z\right\|}_{*}$ denotes the nuclear norm, and tr(Z) denotes the trace of matrix ***Z***. $U\Sigma V^{T}$ denotes the SVD of ***Z***, where $U=\left(u_{1},\ldots ,u_{a}\right)\in R^{a\times a}$, $\Sigma \in R^{a\times b}$, and $V=\left(v_{1},\ldots v_{b}\right)\in R^{b\times b}$. $I_{r}$ denotes a *r*-order identity matrix.

## 4. System Model

This section will show how to use the CSI collected from the network interface card (NIC) to estimate the moving object’s location in the indoor environment.

### 4.1. System Architecture

**Figure 1:**
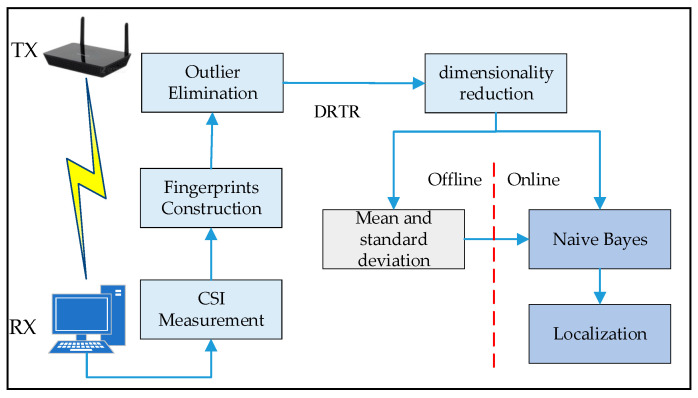
System architecture.

Figure 1 shows the architecture of the SICD system. The SICD is composed of two stages, namely the offline stage and online stage. In the offline stage, we first collect the data of CSI and construct localization fingerprints. Then, the dimension of preprocessed data is reduced by leveraging the algorithm of dimensionality reduction based on locally linear embedding (LLE) [24,25,26,27] and low rank. Finally, the mean and standard deviation of fingerprints at each location are calculated. In the online stage, we use naive Bayes classifier to predict the location of the moving object and output the result of localization.

### 4.2. CSI Data Collection

To collect CSI data, we selected the TP-LINK wireless router with two antennas as the access point (AP) and the desktop computer with the NIC5300 as the terminal. Moreover, the wireless network card has three antennas. We placed the AP and terminal at two ends of the test area, respectively. However, the acquisition of CSI data must meet the hardware mentioned above conditions and requires the use of a software collection tool based on Linux 802.11n, namely, the CSI TOOL [28].

**Figure 2:**
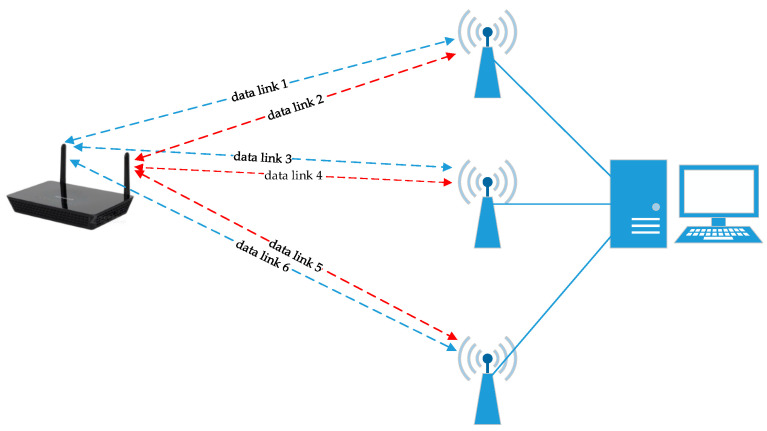
CSI-MIMO antenna pairs. MIMO: multiple-input multiple-output.

**Figure 3:**
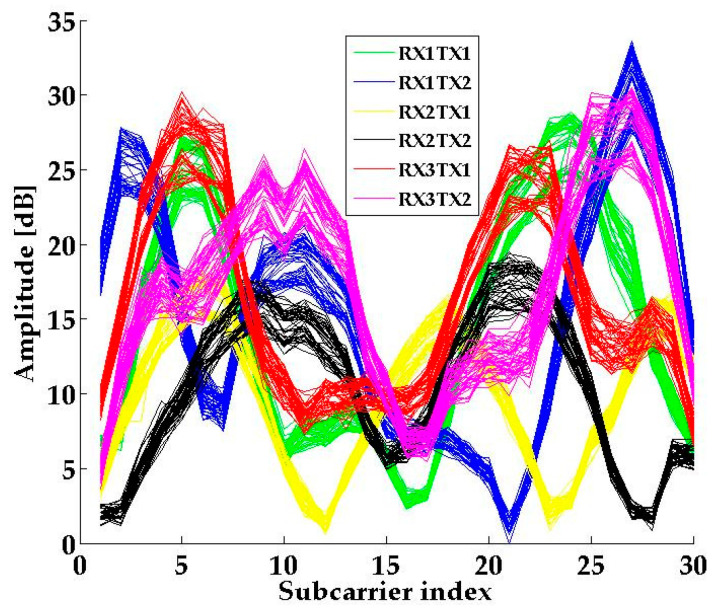
The CSI amplitude pattern of different antenna pairs.

The CSI TOOL is used to analyze the data which are received by the terminal. The data contains various types of information, e.g., the rate, the number of received and transmitted antennas, and the CSI. For simplicity, we selected CSI amplitude as the data feature. The traditional localization method based on CSI-MIMO requires multiple APs or terminals to construct the MIMO system [29,30,31]. In SICD, we use a single AP and terminal with multiple antennas to construct MIMO system. As shown in Figure 2, each transmitted and received antenna forms a data link. The CSI amplitude patterns of different received and transmitted antenna pairs are different, as shown in Figure 3. If we do not process the CSI amplitudes of all antenna pairs together, much valuable information related to MIMO will be lost.

### 4.3. Localization Fingerprint Construction

We collect *m* data packets of CSI for each sub-carrier from every antenna pair, and then the CSI amplitude matrix of the *i*-th antenna pair is defined as

(9) $${\left|H\right|}_{i}=\left(\begin{array}{ccc} \left|H_{11}\right| & \cdots & \left|H_{1n}\right|\\ \vdots & \ddots & \vdots \\ \left|H_{m1}\right| & \cdots & \left|H_{mn}\right| \end{array}\right).$$

**Figure 4:**
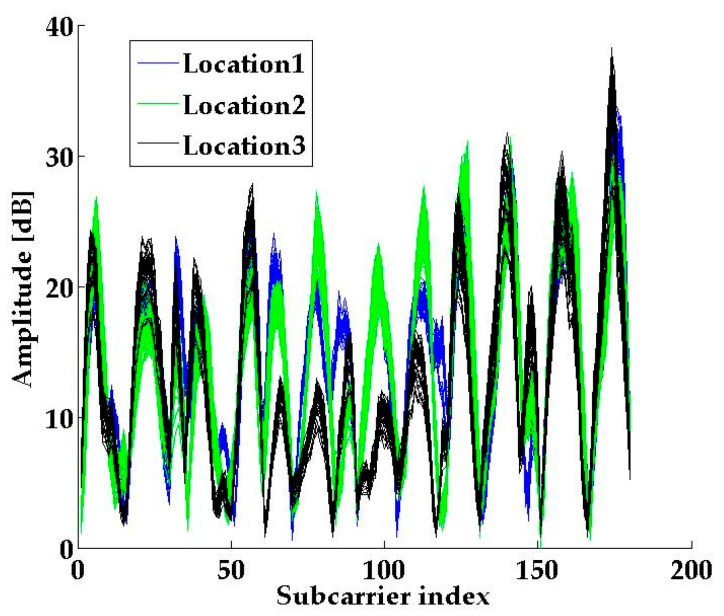
CSI amplitude from three different locations.

In SICD, we combine the CSI amplitude matrix of all antenna pairs as

(10) $$\left|\hat{H}\right|=\left[{\left|H\right|}_{1},\ldots ,{\left|H\right|}_{i},\ldots ,{\left|H\right|}_{g}\right],$$

where *g* denotes the number of antenna pairs. Figure 4 shows the CSI amplitude of 10 packets from three different locations. We can see that the amplitude pattern is quite different in different locations. It means that the CSI-MIMO amplitude fingerprint based on a single AP can be used for indoor localization.

In order to facilitate the data dimensionality reduction processing in the following sub-section, we take *l* of the *m* data packets to form a fingerprint, and arrange them into the form of a one-dimensional vector as

(11) $$\left|\tilde{H}\right|=\left[\left|H_{1,1}\right|,\ldots ,\left|H_{1,n\times g}\right|,\left|H_{2,1}\right|,\ldots ,\left|H_{2,n\times g}\right|,\ldots ,\left|H_{l,n\times g}\right|\right].$$

### 4.4. Data Dimensionality Reduction

#### 4.4.1. Outlier Elimination

**Figure 5:**
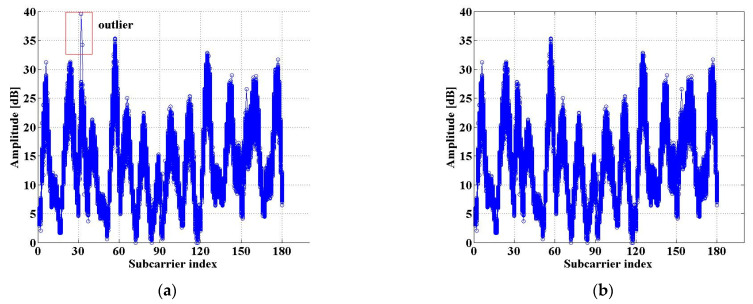
Effectiveness of noise reduction preprocessing: (**a**) The amplitude data of CSI-MIMO before processing; (**b**) The amplitude data of CSI-MIMO after processing.

In the indoor wireless environment, the CSI will be affected by noise. This means that the amplitude data of CSI-MIMO contains some outliers, which are not conducive to the extraction of features for localization from the amplitude data. However, outliers generally deviate significantly from the average. As shown in Figure 5a, the CSI values marked by a red square at sub-carrier 31 and sub-carrier 32 deviate greatly from other values of the two corresponding sub-carriers, respectively. Before reducing the dimensionality of the CSI-MIMO amplitude data, the outliers in the data should be eliminated. We use the Pauta criterion to eliminate outliers in the CSI amplitude data [32,33].

The $\left|\tilde{H}\right|$ is an input sample of Pauta. The mean and standard deviation of $\left|\tilde{H}\right|$ are calculated as

(12) $$\mathrm{mean}=\frac{\sum \left|H_{l,n\times g}\right|}{l\times n\times g},$$

(13) $$std=\sqrt{{\left(\left|H_{1,1}\right|-mean\right)}^{2}+{\left(\left|H_{1,2}\right|-mean\right)}^{2}+\cdots +{\left(\left|H_{l,n\times g}\right|-mean\right)}^{2}}.$$

According to (12) and (13), the outlier decision rule is set as

(14) $$\left|\left|H_{l,n\times g}\right|-mean\right|>3std.$$

According to (14), the output of Pauta algorithm is represented as

(15) $${\left|H_{l,n\times g}\right|}^{*}=\{\begin{array}{c} \mathrm{mean},\left|\left|H_{l,n\times g}\right|-mean\right|>3std\\ \left|H_{l,n\times g}\right|,\left|\left|H_{l,n\times g}\right|-mean\right|\leq 3std \end{array}.$$

After being processed, the amplitude data of CSI-MIMO are improved, with noise greatly suppressed, as shown in Figure 5b.

#### 4.4.2. Dimensionality Reduction Algorithm

To reduce the redundancy in the CSI-MIMO amplitude data and improve localization accuracy, we designed a dimensionality reduction algorithm based on LLE and low-rank (DRLL). The general framework of the DRLL can be formulated as

(16) $$\underset{w}{\mathrm{argmin}}\;L\left(W\right)+\alpha rank\left(W\right)+\beta f\left(W\right),$$

where L(W) is the loss function, and rank(W) is the regularization term used for low-rank constraint. f(W) is the regularization term used to ensure that the points close to each other in the original space after mapping are also close to each other in the new space. $W\in R^{d\times e}$ is the projection matrix. α and β are balance parameters.

In this paper, we use the least square loss function to evaluate the approximate error of data before and after dimensionality reduction. The loss function is defined as

(17) $$\underset{w}{\mathrm{argmin}}\;L\left(W\right)={\left\|Y-XW\right\|}_{F}^{2},$$

where $Y=\;\left[y_{1},y_{2},\ldots ,y_{c}\right]\in R^{c\times e}$ is the data after dimensionality reduction. $X=\;\left[x_{1},x_{2},\ldots ,x_{c}\right]\in R^{c\times d}$ (d≫e) indicates the data before dimensionality reduction. ${\|•\|}_{F}$ represents the Frobenius norm of a matrix.

The rank function is non-convex, and its solution is NP (Nondeterministic polynominal)-hard. Therefore, we impose a low-rank constraint on the projection matrix ***W*** by using the truncated nuclear norm to approximate the rank of the matrix [34,35,36,37]. The truncated nuclear norm is defined as

(18) $${\left\|W\right\|}_{r}=\underset{W}{\min}{\left\|W\right\|}_{*}-\mathrm{tr}\left(CWD^{T}\right).$$

In order to keep the data structure after dimensionality reduction consistent with the raw data, we use locally linear embedding (LLE) as the regularization term.

(19) $$f\left(W\right)=\;\frac{1}{2}{\sum_{i=1}^{c}\left\|y_{i}-\sum_{j=1}^{k}o_{ij}y_{j}\right\|}_{F}^{2}=\;\mathrm{tr}\left(W^{T}X^{T}QXW\right),$$

where

(20) $$Q=\;{\left(I-O\right)}^{T}\left(I-O\right),$$

*k* is the number of nearest neighbors, and *o* is the weight coefficient. ***I*** refers to an c-order identity matrix. ***O*** is the weight matrix, which is composed of the weight coefficient *o*.

According to (16) and (18)–(20), the model of the DRLL algorithm can be expressed as

(21) $$\underset{w}{\mathrm{argmin}}{\left\|Y-XW\right\|}_{F}^{2}+\alpha \left[{\left\|W\right\|}_{*}-\mathrm{tr}\left(CWD^{T}\right)\right]+\beta \mathrm{tr}\left(W^{T}X^{T}QXW\right).$$

#### 4.4.3. Optimal Solution

In order to solve the optimization problem of (21), we use the method of augmented Lagrangian multiplier [38,39] to find the optimal solution, which is shown in detail as follows.

At first, we convert (21) to the following equivalent problem as

(22) $$\underset{w}{\mathrm{argmin}}{\left\|Y-XW\right\|}_{F}^{2}+\alpha \left[{\left\|J\right\|}_{*}-\mathrm{tr}\left(CGD^{T}\right)\right]+\beta \mathrm{tr}\left(W^{T}X^{T}QXW\right)\;s.t.\;W=J,W=G.$$

This problem can be solved by the augmented Lagrangian multiplier method, which aims to minimize the following augmented Lagrangian function of

(23) $$\begin{array}{l} \underset{W,J,G,\eta ,\xi }{\mathrm{argmin}}{\left\|Y-XW\right\|}_{F}^{2}+\alpha \left[{\left\|J\right\|}_{*}-\mathrm{tr}\left(CGD^{T}\right)\right]+\beta \mathrm{tr}\left(W^{T}X^{T}QXW\right)+\frac{\lambda }{2}{\left\|W-J\right\|}_{F}^{2}+\mathrm{tr}\left(\eta^{T}\left(W-J\right)\right)\\ +\frac{\lambda }{2}{\left\|W-G\right\|}_{F}^{2}+\mathrm{tr}\left(\xi^{T}\left(W-G\right)\right) \end{array},$$

where λ>0 is a penalty parameter. η and ξ are the Lagrangian multipliers. We use the alternating iteration method to solve problem (23) by fixing four of the five variables (***W***, ***J***, ***G***, η, and ξ).

When ***J***, ***G***, η, and ξ are fixed, we can optimize ***W*** by

(24) $$\underset{W}{\mathrm{argmin}}{\left\|Y-XW\right\|}_{F}^{2}+\beta \mathrm{tr}\left(W^{T}X^{T}QXW\right)+\frac{\lambda }{2}{\left\|W-J\right\|}_{F}^{2}+\mathrm{tr}\left(\eta^{T}\left(W-J\right)\right)+\frac{\lambda }{2}{\left\|W-G\right\|}_{F}^{2}+\mathrm{tr}\left(\xi^{T}\left(W-G\right)\right).$$

The closed-form solution of optimal $W^{*}$ is represented as

(25) $$W^{*}={\left(2X^{T}X+2\beta X^{T}QX+2\lambda I_{d}\right)}^{-1}\left(2X^{T}Y+\lambda J+\lambda G-\eta -\xi \right),$$

where $I_{d}$ denotes a d-order identify matrix.

To optimize the ***J***, we first need to fix ***W***, ***G***, η, and ξ, and then solve the following problem as

(26) $$\underset{J}{\mathrm{argmin}}\alpha {\left\|J\right\|}_{*}+\frac{\lambda }{2}{\left\|W^{*}-J\right\|}_{F}^{2}+\mathrm{tr}\left(\eta^{T}\left(W^{*}-J\right)\right).$$

The optimal $J^{*}$ is represented as

(27) $$J^{*}=\underset{J}{\mathrm{argmin}}\frac{\alpha }{\lambda }{\left\|J\right\|}_{*}+\frac{1}{2}{\left\|W^{*}-J+\frac{\eta^{T}}{\lambda }\right\|}_{F}^{2},$$

which can be solved by the singular value threshold operator [40].

With the ***W***, ***J***, η, and ξ being fixed, variable ***G*** is optimized by

(28) $$\underset{G}{\mathrm{argmin}}\frac{\lambda }{2}{\left\|W^{*}-G\right\|}_{F}^{2}+\mathrm{tr}\left(\xi^{T}\left(W^{*}-G\right)\right)-\alpha \mathrm{tr}\left(CGD^{T}\right).$$

According to (28), the optimal $G^{*}$ can be calculated by

(29) $$G^{*}=W^{*}+\frac{1}{\lambda }\left(\alpha C^{T}D+\xi \right).$$

At each step, we optimize the Lagrangian multipliers by

(30) $$\eta^{*}=\eta +\lambda \left(W^{*}-J^{*}\right),$$

(31) $$\xi^{*}=\xi +\lambda \left(W^{*}-G^{*}\right).$$

The detailed solution procedures of the DRLL model are listed in Algorithm 1. Moreover, the ADMM (Alternating Direction Multiplier Method) guarantees the convergence of the DRLL model solution [23].
**Algorithm 1. Solution of the DRLL model****Input: *X***, ***C***, ***D***, *k*, α, β, λ, *t*, *e*;**Output:**$W^{*}$; **1** Initialize projection matrix ***W*** as the matrix with orthogonal column vectors, ***W*** = ***J*** = ***G***, η = ξ = ***0***; **2** for T = 1:*t* **3** Optimize ***W*** by        
$W^{*}={\left(2X^{T}X+2\beta X^{T}QX+2\lambda I_{d}\right)}^{-1}\left(2X^{T}Y+\lambda J+\lambda G-\eta -\xi \right)$; **4** Optimize ***J*** by $J^{*}=\underset{J}{\mathrm{argmin}}\frac{\alpha }{\lambda }{\left\|J\right\|}_{*}+\frac{1}{2}{\left\|W^{*}-J+\frac{\eta^{T}}{\lambda }\right\|}_{F}^{2}$; **5** Optimize ***G*** by $G^{*}=W^{*}+\frac{1}{\lambda }\left(\alpha C^{T}D+\xi \right)$; **6** Optimize η by $\eta^{*}=\eta +\lambda \left(W^{*}-J^{*}\right)$; **7** Optimize ξ by $\xi^{*}=\xi +\lambda \left(W^{*}-G^{*}\right)$; **8 end**

### 4.5. Naive Bayes Classification for Localization

The device-free indoor localization based on CSI can be converted into a classification problem to obtain the moving object’s location information. To this end, we use the naive Bayes classification algorithm [41] in machine learning to achieve indoor localization. Naive Bayes classification is a method based on the Bayesian theorem. The method assumes that the characteristic conditions are independent of each other. The Bayesian theorem is defined as

(32) $$P\left(\phi |\delta \right)=\frac{P\left(\delta |\phi \right)\cdot P\left(\phi \right)}{P\left(\delta \right)},$$

where P(·|∗) denotes the conditional probability, and P(·) denotes the probability of the event.

According to (32), the naive Bayes classifier in SICD is formulated as

(33) $$P\left(Lc_{i}|Ft\right)=\frac{P\left(Ft|Lc_{i}\right)\cdot P\left(Lc_{i}\right)}{P\left(Ft\right)},$$

where $Lc_{i}$ represents *i*-th location point, and Ft represents the feature to be classified. In order to predict the location of the moving object, we choose the location point of the category with the highest probability as the final location of the moving object, which can be calculated by

(34) $$Lc\leftarrow \mathrm{argmax}\;P\left(Lc_{i}|Ft\right)=\;\mathrm{argmax}\frac{P\left(Ft|Lc_{i}\right)\cdot P\left(Lc_{i}\right)}{P\left(Ft\right)}.$$

Since $P\left(Lc_{i}\right)$ and P(Ft) are known, (34) can be written equivalently as

(35) $$\mathrm{argmax}\;P\left(Lc_{i}|Ft\right)=\;\mathrm{argmax}\;P\left(Ft|Lc_{i}\right).$$

To calculate the maximum value of $P\left(Ft|Lc_{i}\right)$, we assume that it obeys the log-normal distribution as

(36) $$P\left(Ft|Lc_{i}\right)=\frac{1}{{\left[2\pi {\left(STD\right)}^{2}{\left(Ft\right)}^{2}\right]}^{1/2}}\exp\left[-\frac{{\left(\ln\left(Ft\right)-\mu \right)}^{2}}{2{\left(STD\right)}^{2}}\right],$$

where ${\left(STD\right)}^{2}$ and μ denote the variance and mean, respectively.

## 5. Experiment Validation

The implementation and performance evaluation of SICD are introduced in this section. The environment of the experiment is first described. Then, we analyze the influence of setting different experimental parameters on the SICD. Finally, we compare the SICD with other off-the-shelf methods.

### 5.1. Experimental Setup

In the performance verification experiment of SICD, we used a single TP-link wireless router as the access point. The model of the router was TL-WR840N, which carries two antennas. A Lenovo M736E desktop computer with the NIC5300 was used as the terminal. A ThinkStation P720 server with NIVIDA Quadro P2200 Graphic Card worked on predicting location. Table 1 shows the system configuration of measurements.

**Figure 6:**
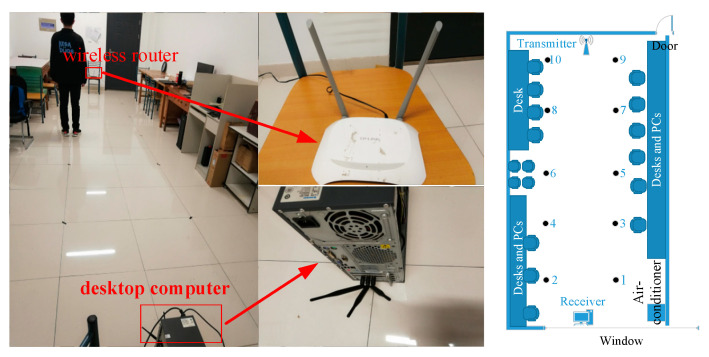
Experimental environment and equipment.

The CSI measurement experiments were conducted in the laboratory of Nanjing University of Posts and Telecommunications. The laboratory has some obstacles, such as chairs, tables, and computers. Simultaneously, we selected 10 locations to train and test in an experimental area of approximately $3\times 6.8\;m^{2}$. Figure 6 reveals the environment of the CSI measurement experiments and the equipment required for the measurement. When the moving object is located in different indoor locations, the terminal receives the data packets from the AP. During the data collection, the moving object does not change his posture. The AP sent data packets with the interval of 0.01 s, and we collected 30,000 data packets at each location. We divided the entire data sets into training set and test set with a ratio of 7:3.

### 5.2. Convergence and Complexity of DRLL

#### 5.2.1. Convergence of DRLL

**Figure 7:**
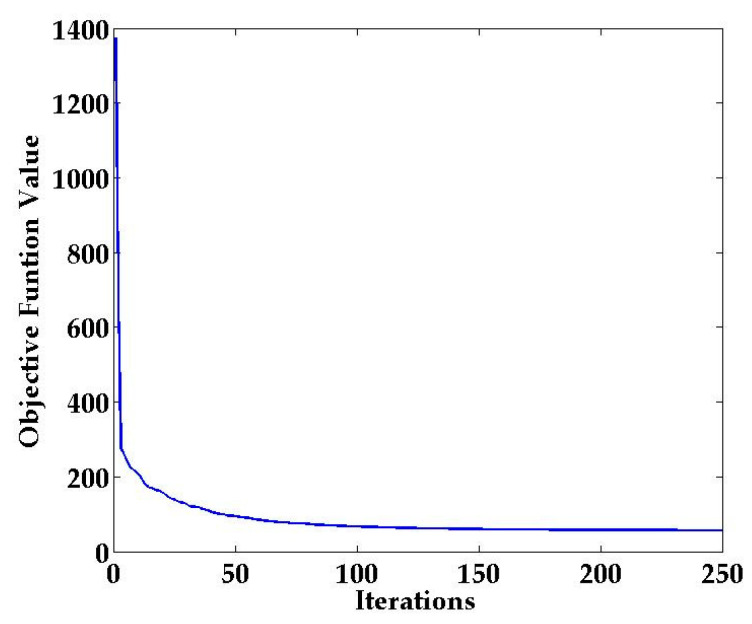
Convergence of the dimensionality reduction algorithm based on locally linear embedding (LLE) and low rank (DRLL).

To study the convergence of the DRLL algorithm, we calculated the value of (22) by leveraging the CSI amplitude data, as shown in Figure 7. From Figure 7, we can observe that the value of the objective function gradually decreases as the number of iterations increases, and gradually converges after a certain number of iterations. This indirectly explains that our DRLL algorithm is feasible.

#### 5.2.2. Complexity of DRLL

In order to study the complexity of DRLL, we can decompose it into five equation: Equation (25), Equation (27), Equation (29), Equation (30), and Equation (31). For each iteration, Equation (25) needs to construct $\left(2X^{T}X+2\beta X^{T}QX+2\lambda I_{d}\right)$ and $\left(2X^{T}Y+\lambda J+\lambda G-\eta -\xi \right)$, which will cost $O\left(d^{3}+d^{2}c+dc^{2}+edc\right)$. The main cost of Equation (27) is to calculate the singular value threshold operator for which the complexity is $\min\left\{O\left(de^{2}\right),O\left(d^{2}e\right)\right\}$. The complexity of Equation (29) is O(de). The complexity of Equation (30) and Equation (31) is O(de), because they only need matrix addition and subtraction. In summary, the complexity of DRLL is $O\left(d^{3}+d^{2}c+dc^{2}+de^{2}\right)$.

### 5.3. Analysis of Parameter Setting

#### 5.3.1. Impact of Number of Nearest Neighbors

In DRLL, we use LLE to ensure that the data structure after dimensionality reduction is consistent with the raw data. To verify the impact of the *k* nearest neighbors (KNN) in the LLE on the proposed SICD localization method, we conducted a specific experiment with different *k* values.

**Figure 8:**
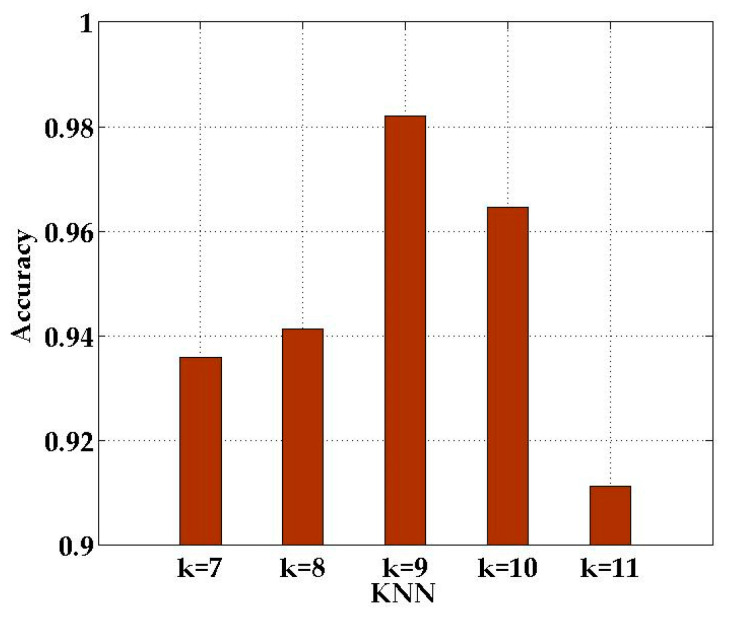
Comparison of nearest neighbor in different numbers.

Figure 8 reveals the recognition rate of SICD with different number of nearest neighbors at each location. Figure 8 shows that when *k* = 9, SICD has the highest recognition rate at most locations. Thus, we set the number of nearest neighbors to nine.

#### 5.3.2. Impact of Number of Data Packets in the Sample

In the experiments, we used 3*2 MIMO technology and combined different antenna pairs of each received antenna according to (11). In order to evaluate whether the number of CSI data packets contained in each sample had an impact on the SICD localization method, we used the amplitude information carried by 30, 60, 90, 120, and 150 data packets to construct the respective fingerprint samples.

**Figure 9:**
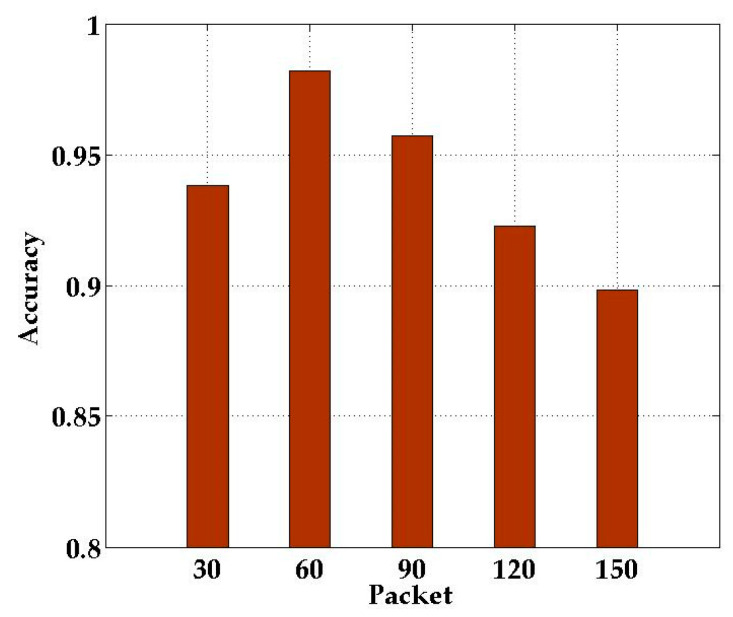
Comparison of data packet in different numbers.

Figure 9 shows the recognition rate of SICD with different numbers of data packets in the sample. As shown in Figure 9, when the number of data packets constituting the sample was 60, SICD had the highest recognition rate. Therefore, we chose 60 CSI data packets to construct the localization fingerprint.

#### 5.3.3. Impact of the Dimension of Classification Samples

In order to verify the impact of the dimensionality of the classification samples on the localization effect, we first leveraged the proposed DRLL algorithm to reduce the dimension of the CSI amplitude data to 60, 120, 180, and 240, respectively. Then, these dimensionality-reduced samples were input into the classifier for localization verification.

**Figure 10:**
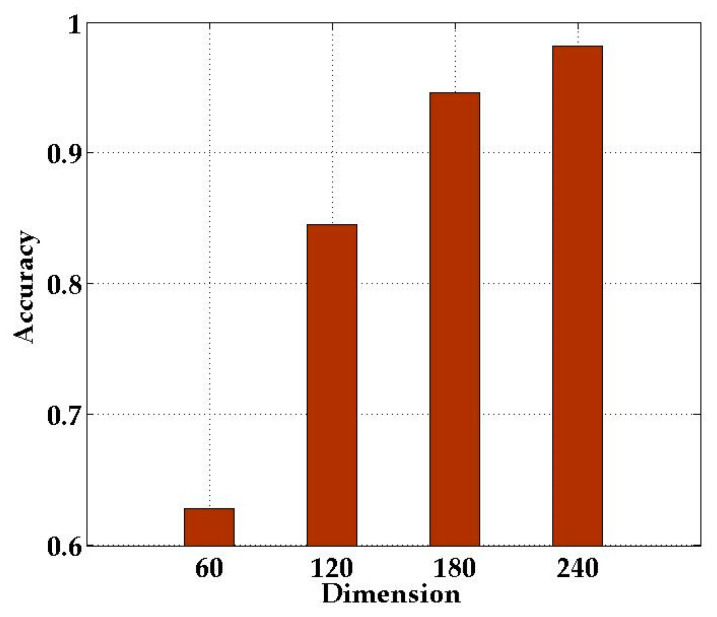
Comparison of classification sample in different dimensions.

Figure 10 presents the recognition rate of different classification sample dimensions. The samples with dimension 240 achieved the highest recognition rate. The above experimental results show that satisfactory localization recognition rate could be obtained by reducing the CSI amplitude data to 240.

#### 5.3.4. Impact of Conditional Probability Distribution

When we calculate the conditional probability in the naive Bayes classifier, it needs to obey the log-normal distribution. In order to study the impact of the conditional probability distribution on the localization effect, we compared the localization accuracy of conditional probability which obeyed the log-normal distribution and Gaussian distribution, respectively.

**Figure 11:**
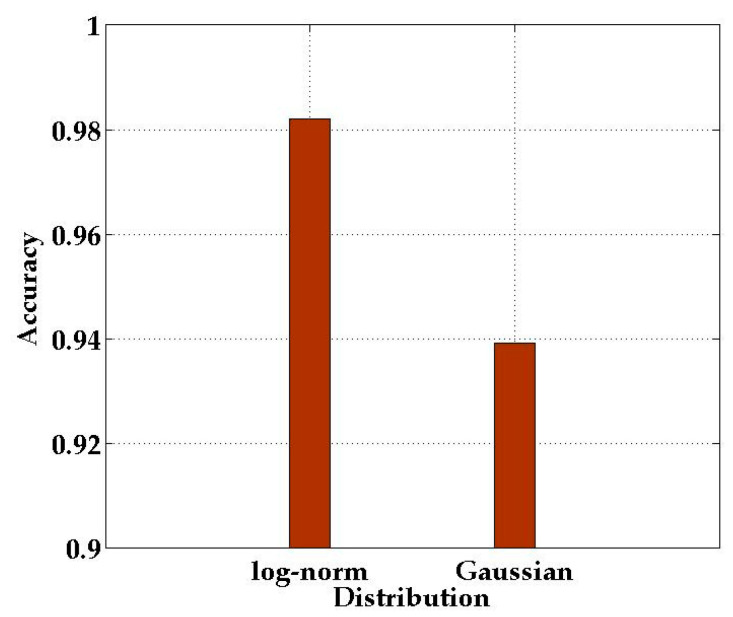
Comparison of conditional probability under different distributions.

As shown in Figure 11, when the conditional probability obeys the Gaussian distribution, the recognition rate is lower than the log-normal distribution. Based on the above phenomenon, we can conclude that when the conditional probability obeys log-normal distribution, the localization performance of SICD can be greatly facilitated.

### 5.4. Comparison with Existing Dimensionality Reduction Algorithms

We verified the performance of our DRLL algorithm and compared it with three existing dimensionality reduction algorithms: LLE [24], PCA (Principal Component Analysis) [42], and NPE (Neighborhood Preserving Embedding) [43]. We used the recognition rate to evaluate the performance of the four algorithms.

**Figure 12:**
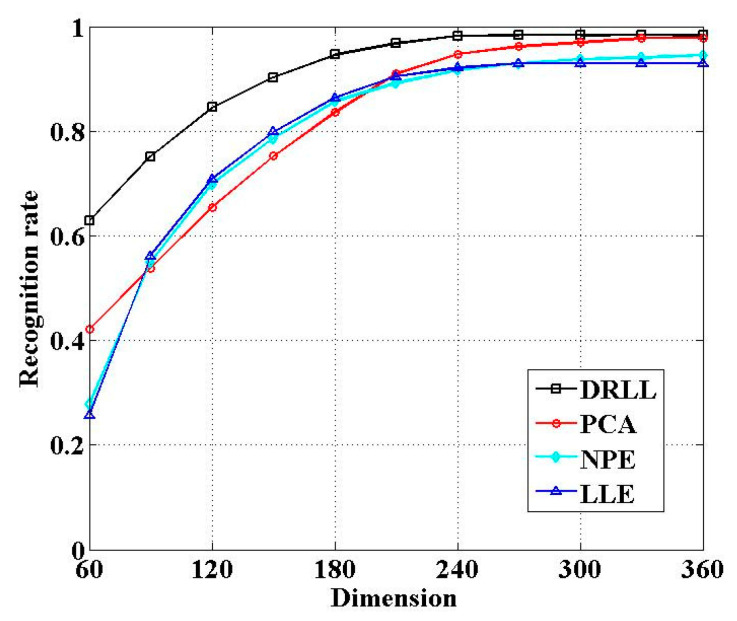
Comparison of four algorithms under varying dimensions of samples.

Figure 12 shows the variation of the recognition rate of four dimensionality reduction algorithms in different dimensions. With the increase in sample dimensions after dimensionality reduction, the recognition rate gradually increased. When the dimension was 240, the recognition rate of DRLL could reach 98.2%. We could clearly observe that the performance of the DRLL algorithm was better than that of the other three algorithms.

### 5.5. Comparison with Existing Localization Methods

We verified the performance of our SICD method and compared it with three state-of-the-art CSI-based localization methods, ConFi [44], PICN [32], and MaLDIP [45]. ConFi is based on the theory of deep learning. PICN and MaLDIP are based on the Bayes and support vector machine (SVM) theory, respectively. Here, the number of training samples of the four localization methods is the same, and the number of test samples is also the same.

Table 2 records the localization errors of the four methods mentioned above. The mean and standard deviation (STD) of SICD error are 1.3730 m and 0.3762 m, respectively. It can be clearly seen from the table that our SICD localization method is better than the other three methods.

**Figure 13:**
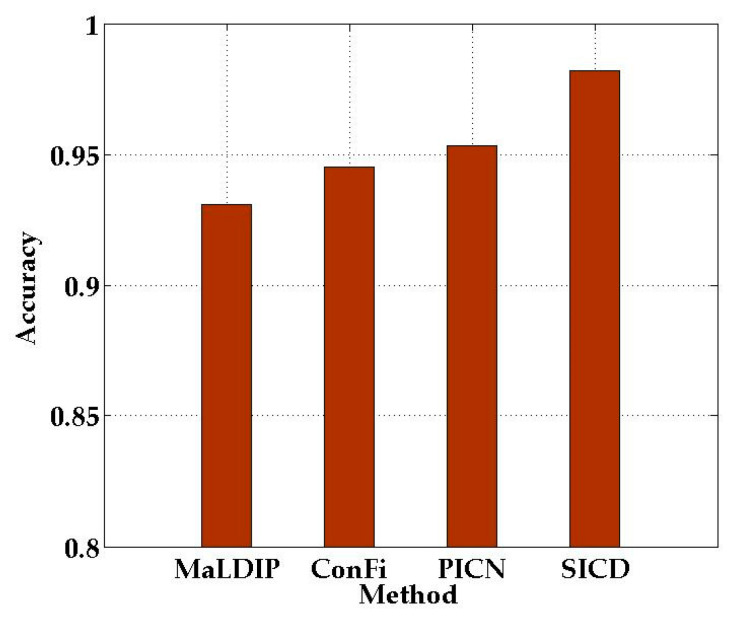
Average localization accuracy of the different methods.

The average localization accuracy of the four methods is presented in Figure 13. The average localization accuracy of our proposed SICD localization method is as high as 98.2%, which is significantly higher than that of the other three localization methods. We can conclude that SICD outperforms the other three methods, which confirms the effectiveness of our method.

**Figure 14:**
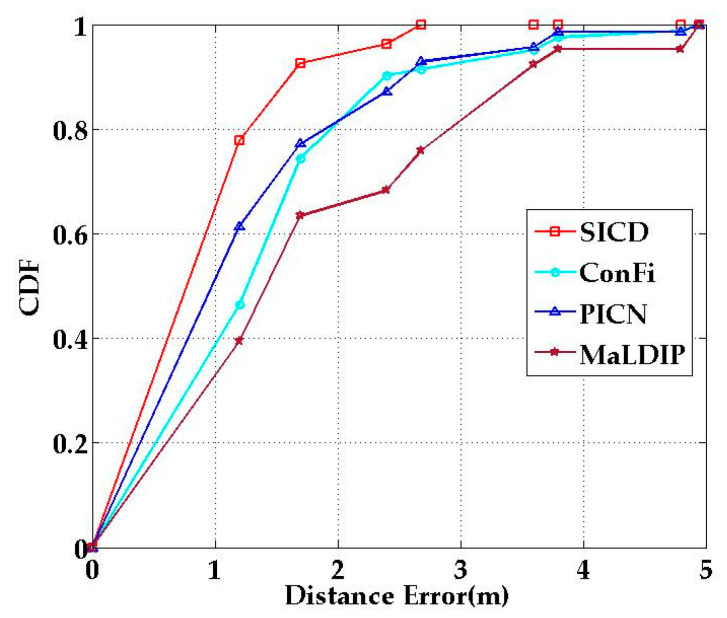
Cumulative distribution function (CDF) of localization errors with different methods.

Figure 14 shows the cumulative distribution function (CDF) of error distance with the four localization methods. It can be seen from the figure that the CDF curve of SICD is at the top of all curves. As can be seen from Figure 14, with our SICD method 92.59% of the test data had a localization error of less than 2 m, in comparison to 63.46% for MaLDIP, 74.39% for ConFi, and 77.14% for PICN Therefore, we can conclude that the SICD method had the best performance among these four localization methods.

## 6. Conclusions

In this paper, we proposed the SICD, a single-access-point indoor localization method based on CSI-MIMO with dimensionality reduction. In the SICD we used a single access point to measure the CSI and constructed a fingerprint with rich localization information. In order to reduce the redundant information in fingerprint data, we designed an effective dimensionality reduction algorithm, namely the DRLL. In addition, we leveraged the log-normal distribution to calculate the conditional probability in the naive Bayes classifier, improving the classification performance of the classifier. Extensive experimental results showed that the SICD achieved a localization accuracy of 98.2% in the mess laboratory.

## 7. Future Work

In this paper, we provided a unique understanding of CSI-MIMO based localization and established a robust model. In the next step, we will focus on identifying the location of multiple moving objects indoors. Specifically, the multi-link of CSI-MIMO will be used to identify and extract the path reflection corresponding to each moving object. The location fingerprint of each moving object will be further constructed as if there was only a single object in the environment to promote the localization of multiple moving objects.

## Figures and Tables

**Table 1:** System configuration of measurements.

Parameter	Value
Transmitted antenna height	0.45 m
Received antenna height	0.06 m
Number of transmitted antennas	2
Number of received antennas	3
Bandwidth	20 MHz
Center frequency	2.4 GHz
Number of subcarriers	30

**Table 2:** Localization errors with CSI-based methods.

Method	Mean Error (m)	STD (m)
SICD	1.3730	0.3762
PICN	1.6791	0.7979
ConFi	1.7885	0.8261
MaLDIP	2.1386	1.1081

## Data Availability

All data included in this study are available upon request by contact with the corresponding author.
